# Editorial: Lipid alterations in cancer development, resistance and recurrence

**DOI:** 10.3389/fcell.2024.1493626

**Published:** 2024-09-27

**Authors:** F. Pagliari, S. Di Franco, L. Tirinato

**Affiliations:** ^1^ Division of Biomedical Physics in Radiation Oncology, German Cancer Research Center (DKFZ), Heidelberg, Germany; ^2^ Department of Precision Medicine in Medical, Surgical and Critical Care, University of Palermo, Palermo, Italy; ^3^ Department of Medical and Surgical Sciences, University Magna Graecia, Catanzaro, Italy

**Keywords:** lipids-metabolism, lipids, cancer, metabolism, lipid droplet

Lipids, as well as proteins and nucleic acids, are essential for mammalian cellular biology, as they play critical roles in cellular membrane architecture, in intra- and intercellular signaling, and as fundamental energy storage. These molecular mechanisms are particularly pronounced in the context of tumors, where neoplastic cells must adapt to changes in the microenvironment, characterized by inadequate vascularization, nutrient deprivation, extracellular matrix accumulation resulting from stroma cell recruitment including immune cell infiltration, and the expansion of cancer-associated fibroblasts (CAFs).

Transformed cells rely on both lipid internalization and the mobilization of intracellular lipid reservoirs, through several mechanisms such as *de novo* lipid biosynthesis, the mobilization of lipid droplets (LDs), and membrane remodeling. Additionally, lipid mediators, such as prostaglandin E2, cholesterol, and fatty acids derived from either the stroma or the tumor tissue, may facilitate the recruitment of non-malignant host cells, including immune cells and CAFs.

Despite the complexity involved, these aspects underscore the necessity of a deeper understanding of the functions that the different lipid molecules and their metabolism play in cancer development, as well as in its progression and resistance to therapies.

In this Research Topic “Lipid Alterations in Cancer Development, Resistance and Recurrence”, the study of Gu et al. aimed to determine the effects of lipid metabolism on the progression and development of hepatocellular carcinoma (HCC). The authors used the expression profiles and clinical data of 371 and 231 patients with HCC obtained from the TCGA and Internal Cancer Genome Consortium (ICGC) databases, respectively. Through Cox and LASSO regression analyses, a prognostic risk model was constructed based on the lipid metabolism-associated genes (LMAG) data. The tumor mutation burden (TMB), immune cell infiltration levels, and immune response checkpoints of the identified risk groups were determined and compared. A total of two clusters were identified based on the LMAG expression, showing significant differences in tumor stage and immune cell infiltration. A prognostic risk model based on four LMAGs was constructed and proven to have a significant prognostic value. The 1-, 3-, and 5-year survival rates in the high-risk group were 62.2%, 20.5%, and 8.1%, respectively, whereas those in the low-risk group were 78.9%, 28.1%, and 13.5%, respectively. The survival differences between the two risk groups were likely associated with TP53 mutation status, TMB score, degree of immunocyte infiltration, and immune checkpoint level. Likewise, the expression level of every LMAG included in the model had the same effect on the overall survival and immune cell infiltration levels. More importantly, the prognostic value of the signature was verified in an independent ICGC cohort. Thus, the expression levels of LMAGs are closely related to the tumor microenvironment in HCC and may serve as promising biological indicators for prognosis and immune therapy in patients with HCC.

Interestingly, the review written by Maurotti et al. focuses on the role of the LDs in the hepatocarcinoma development. This is of particular importance because, contrary to what believed until some years ago, LDs are now considered a “metabolic hallmark” within many solid tumors like colon, prostate, breast, lung and even neurological tumors. In this review, the authors try to elucidate the LD function in all pre-tumoral stages, including Metabolic Dysfunction-Associated Steatotic Liver Disease (MASLD), Metabolic dysfunction-Associated Steatohepatitis (MASH), and fibrosis, which ultimately contribute to the actual development of the tumor. Some of the most important genetic alterations, as PNPLA3, TM6SF2, MBOAT7, SUGP1, MARC1, and GCKR, responsible for the different pathological liver stages are also examined.

Moving to lung carcinoma, Cole-Skinner et al. have analyzed datasets from The Cancer Genome Atlas (TCGA, n = 542) and the Clinical Proteomic Tumor Analysis Consortium (CPTAC, n = 115) to explore the interplay between lipid metabolism and High Mobility Group Box one protein (HMGB1) in non-small cell lung cancer (NSCLC) tumors. Previous researches have indicated that unsaturated fatty acids can influence the expression of proteins that play a role in tumorigenesis. Here, the study is focused on specific proteins within the lipogenic and HMGB1/RAGE signaling pathways. The results demonstrated a correlation between stearoyl-CoA desaturase 1 (SCD1) and HMGB1 protein levels in NSCLC tumors. Additionally, *in vitro* experiments revealed that modulating SCD1 activity resulted in alterations in HMGB1 release, which was associated with changes in PD-L1 expression on the surfaces of lung cancer and innate immune cells. Preliminary analyses of select NSCLC patient samples indicated a potential association between serum HMGB1 levels and tumor-associated PD-L1 expression.

In order to discover new strategies of treating cancer, it is necessary to develop a profound knowledge of the mechanisms through which disrupted lipid metabolism impacts the processes of cancer progression. It is clear that targeting a pathway or only a single-step of lipid metabolism will be probably insufficient in an attempt to develop more efficient cancer therapies. Combinatorial therapeutic approaches aimed at inhibiting tumor growth while enhancing the capacity of the immune system to target cancer cells seem to offer promising opportunities in the pharmacological research of lipid-based agents. However, there is currently limited knowledge about how oncogenic signaling pathways indirectly affect the cancer microenvironment and how they can influence the lipid metabolic processes. In their review, Khan et al. tried to elucidate the complex interactions between lipid reprogramming and signaling pathways in the context of cancer. Indeed, the authors suggest a valuable strategy for developing anticancer treatments through the identification of metabolic inhibitors. Furthermore, the review encompasses a comprehensive overview of pharmacological agents that specifically target lipid metabolism in cancer.

In conclusion, the articles included in this Research Topic address current and future questions while highlighting several important functions that lipids can play in various aspects of cancer.

